# Clinicopathological Correlation of Chronic Thromboembolic Pulmonary Hypertension: A Retrospective Study

**DOI:** 10.3390/jcm11226659

**Published:** 2022-11-10

**Authors:** Ziyi Chang, Jixiang Liu, Bei Wang, Honglei Zhang, Ling Zhao, Yunchao Su, Wanmu Xie, Qiang Huang, Yanan Zhen, Fan Lin, Min Liu, Qian Gao, Wenyi Pang, Zhu Zhang, Han Tian, Yishan Li, Peiran Yang, Zhenguo Zhai, Dingrong Zhong

**Affiliations:** 1 Department of Pathology, China-Japan Friendship Hospital, Beijing 100029, China; 2 Department of Pulmonary and Critical Care Medicine, Center of Respiratory Medicine, China-Japan Friendship Hospital, Beijing 100029, China; 3 National Clinical Research Center for Respiratory Diseases, Beijing 100029, China; 4 Graduate School of Peking Union Medical College, Chinese Academy of Medical Sciences, Beijing 100730, China; 5 Department of Cardiovascular Surgery, China-Japan Friendship Hospital, Beijing 100029, China; 6 Department of Radiology, China-Japan Friendship Hospital, Beijing 100029, China; 7 The First Clinical Medical College, Shanxi Medical University, No. 56 Xinjiannan Road, Taiyuan 030012, China; 8 Department of Physiology, State Key Laboratory of Medical Molecular Biology, Institute of Basic Medical Sciences, Chinese Academy of Medical Sciences, School of Basic Medicine, Peking Union Medical College, Beijing 100005, China

**Keywords:** chronic thromboembolic pulmonary hypertension, pathology, persistent pulmonary hypertension, atherosclerosis, pulmonary endarterectomy

## Abstract

The pathophysiology of chronic thromboembolic pulmonary hypertension (CTEPH) is largely unknown. Although pulmonary endarterectomy (PEA) is potentially curative, inoperable patients and persistent pulmonary hypertension (PH) following surgery remain a significant problem. In this study, we aim to describe the histopathological characteristics of CTEPH and explore the potential relationship between pulmonary arterial lesions, radiological parameters, and clinical manifestations. Endarterectomized tissues from 81 consecutive patients of CTEPH were carefully collected, sectioned, and examined by experienced pathologists. Pertinent clinical and radiological data were obtained from medical records and operative reports. Neointima, fresh/organized thrombi, recanalized regions, and atherosclerotic lesions were microscopically examined as previously described. Thrombi and atherosclerosis were dominant in UCSD classification level I PEA materials, while recanalized neo-vessels were more frequently observed in UCSD classification level III cases. Degenerative changes of the extracellular matrix were also noticed in the vascular bed. Atherosclerotic lesions were more frequently observed in cases with higher ratio of the pulmonary artery diameter to ascending aorta diameter (PA/AA) reflected by computed tomographic pulmonary arterial scanning. Furthermore, the removal of pulmonary artery complex lesions (with the combination of three to four types of lesions) by PEA was associated with lower postoperative mean pulmonary arterial pressure (mPAP) and decreased incidences of persistent PH. Our study demonstrates that the histopathological features of CTEPH are strongly linked with clinical manifestations and the postoperative outcome after PEA. These data may provide possible evidence for further studies in searching for appropriate causal factors underlying this disease.

## 1. Introduction

Chronic thromboembolic pulmonary hypertension (CTEPH) is considered as a result of unresolved organized thrombus in proximal pulmonary arteries after acute pulmonary embolism (PE) and is accompanied with a secondary vasculopathy, leading to increased pulmonary vascular resistance (PVR) and progressive right heart failure [1]. CTEPH is reported to affect 20% of patients hospitalized by pulmonary hypertension and may be underdiagnosed at the early stages [2]. In addition to lifelong anticoagulation and pulmonary hypertension (PH)-targeting drugs, pulmonary endarterectomy (PEA) and balloon pulmonary angioplasty (BPA) are highly recommended modalities for operable and inoperable patients, respectively, as both of which offer great symptomatic and prognostic improvements with low acute mortality [3].

During the PEA procedure, the fibrotic organized thromboembolic tissue is carefully excised, following the inside of the artery down to lobar, segmental, or subsegmental branches [1,4]. Efforts have been made to investigate the pathology and pathophysiology of CTEPH by assessing the histomorphometric features of pulmonary endarterectomized tissues. In 2002, thromboembolic lesions of CTEPH were, for the first time, classified into four types through visualization upon PEA surgery, according to the presence of fresh thrombus and the distribution of fibrosis or organized thrombus from the main pulmonary artery to distal branches [5]. In a clinicopathologic study of 200 consecutive PEA cases, fibrinous and organized clots, myxoid matrix, cholesterol clefts, and recanalized thrombi were described [6]. Quarck et al. analyzed major vessel lesions of CTEPH and characterized 103 specimens with four kinds of lesions: neointima formation, atherosclerotic lesions, thrombotic lesions, and recanalized regions [7]. Studies have shown that the presence of recanalization is significantly associated with lower post-operative PVR and can be a potential predictor of clinical outcomes [7,8]. However, investigations on the link between other histological lesions and the hemodynamic parameters before and after PEA have been scarce.

In this study, we morphometrically described the pathological characteristics of removed specimens from CTEPH patients who underwent PEA and sought to investigate the possible relationship between major vessel lesions and clinical manifestations.

## 2. Material and Methods

### 2.1. Patient Selection

The study was carried out at China–Japan Friendship Hospital, a national referral center for CTEPH in China. Between December 2016 and March 2021, 272 patients were diagnosed with CTEPH based on well-defined standards [1]. Eighty-one patients underwent bilateral PEA by a multidisciplinary team, according to previously established guidelines [9].

### 2.2. Clinical Assessment

Clinical information was extracted from their medical records. For each patient, age at the time of surgery, sex, heart function, pulmonary artery pressure (PAP), and documented history of acute pulmonary embolism/deep vein thrombosis (PE/DVT) and thrombophilia were carefully recorded. Contrast-enhanced computed tomographic pulmonary arterial scanning (CTPA) was performed on patients after admission; the pulmonary artery diameters (PA) and ascending aorta diameters (AA) were measured at the level of the bifurcation of the pulmonary trunk on the axis view to calculate PA/AA. Right heart catheterization measurement was obtained at baseline (pre-PEA) and 1–3 days after PEA in the intensive care unit prior to removal of the intraoperative Swan-Ganz catheters (post-PEA). PVR was calculated by mean pulmonary arterial pressure (mPAP)- mean pulmonary capillary wedge pressure (mPCWP)/cardiac output (CO) multiplied by 80 for dynes·s^−1^·cm^−5^. Persistent PH was defined as mPAP ≥ 30 mmHg after PEA before patients were extubated [10].

### 2.3. Morphometric Analysis of PEA Specimens

The UCSD classification system was used to describe the macroscopical characteristics of PEA specimens according to the anatomical location: level 0—no evidence of thromboembolic disease, level I—thromboembolic disease starts in the main pulmonary arteries (IC, with complete occlusion of one lung), level II—thromboembolic disease starts at the level of lobar or intermediate pulmonary arteries, level III—thromboembolic disease starts at segmental pulmonary arteries, and level IV—thromboembolic disease starts at subsegmental branches only [3]. The most proximal disease determined the final type if there was a discrepancy between the left and right specimens. Fresh pulmonary endarterectomized tissues were fixed in 10% phosphate-buffered formalin overnight and embedded in paraffin prior to further pathological examination. Cross-sections (3 μm thick) were sampled from the proximal to distal levels of all pulmonary artery branches and were deparaffinized and rehydrated before being stained with hematoxylin–eosin. Elastic and collagen fibers were labeled with Verhoeff Van Gieson staining and Masson’s trichrome staining, respectively. Immunohistochemistry staining of desmin, α-smooth muscle actin (α-SMA), CD31, and CD61 was performed to detect smooth muscle cells, myofibroblasts, endothelial cells, and thrombus formation. All sections were analyzed under a conventional bright field microscope (Nikon Y-THM). Images were captured through a Logene CMOS camera. Major vessel lesions were determined according to a previous report [7]: fresh, old, or newly organized thrombi were labeled by CD61; neointima formation was characterized by the presence of α-SMA-positive cells; atherosclerosis was marked by a necrotic core with foam cells and cholesterol cleft; and recanalized lesions were featured with multiple secondary lumens in the occluded fibrinous region. The combinations of different types of lesions were defined as simple (one to two types) and complex (three to four types) lesions.

### 2.4. Statistical Analysis

Statistical analyses were performed using GraphPad Prism 8.0 software (GraphPad Software, San Diego, CA, USA). Data were presented as medians with first and third quartiles (Q1–Q3), means ± standard deviations (SDs), or absolute numbers and percentages of patients. If data were normally distributed and with similar variances, the Student’s *t*-test (parametric) was used to compare the difference between the two groups. Nonparametric chi-square tests or Fisher exact tests were used to compare proportions. Results with *p* < 0.05 were considered statistically significant.

## 3. Results

### 3.1. Study Population Characteristics and Surgical Outcome

The clinical characteristics of the 81 CTEPH patients were summarized in Table 1. Different from the equal distribution by sex documented for the USA and Europe, and a 75% female preponderance of CTEPH in Japan [9,11], 66.67% of the patients in our study population were male. The median age was 53, which was 5–10 years younger than that in earlier reports [11]. Thrombophilia was discovered in eight patients (9.88%), including antiphospholipid antibody syndrome (seven patients) and antithrombin III deficiency (one patient). Fifty-nine patients (72.84%) had a documented history of acute PE/DVT. The median time from the onset of PH symptoms to PEA (disease duration) was 26 months (range from 4 months to 15 years). More than half of the population (51 patients, 62.96%) presented World Health Organization (WHO) functional class III or IV. Forty-four patients (54.32%) were subjected to at least one pulmonary vasodilator therapy (Ambrisentan, Bosentan, Tadalafil, or Riociguat) before surgery. 

PEA significantly reduced the mPAP (45 ± 11 vs. 26 ± 7 mmHg, *p* < 0.0001) and PVR (937 ± 414 vs. 470 ± 172 dynes·s^–1^·cm^–5^, *p* < 0.0001) levels in CTEPH patients. Perioperative mortality was observed in 4.94% of our study population due to heart failure (two patients), lung reperfusion oedema (one patient), and sepsis (one patient).

### 3.2. Morphometric Analysis of Thromboembolic Disease

All 81 patients were found to have bilateral involvement of thromboembolic lesions. Pulmonary endarterectomized tissues were categorized based on UCSD classification as level I (54.32%), level II (34.57%), and level III (11.11%), whereas level IV disease was not present in our study population (Figure S1 in the Supplementary Materials). Sections of the PEA pulmonary arterial tissue from all 81 CTEPH patients were carefully analyzed under a light microscope. 

**Figure 1:**
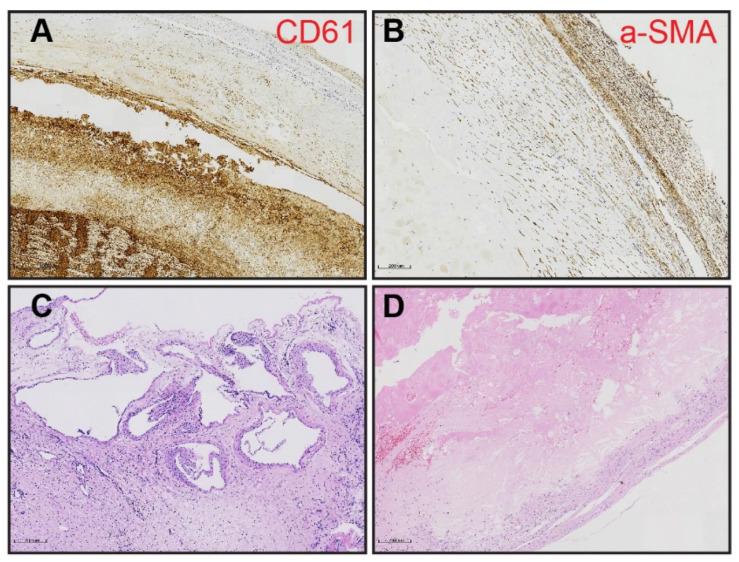
Four basic lesions on the pulmonary vascular bed of CTEPH patients. (**A**) Thrombotic lesions indicated by CD61. (**B**) Neointima formation marked by α-SMA. (**C**,**D**) Atherosclerosis plaque (**C**), and recanalized obstructive lesion (**D**) showed by HE staining. Scale bar = 200 µm.

**Figure 2:**
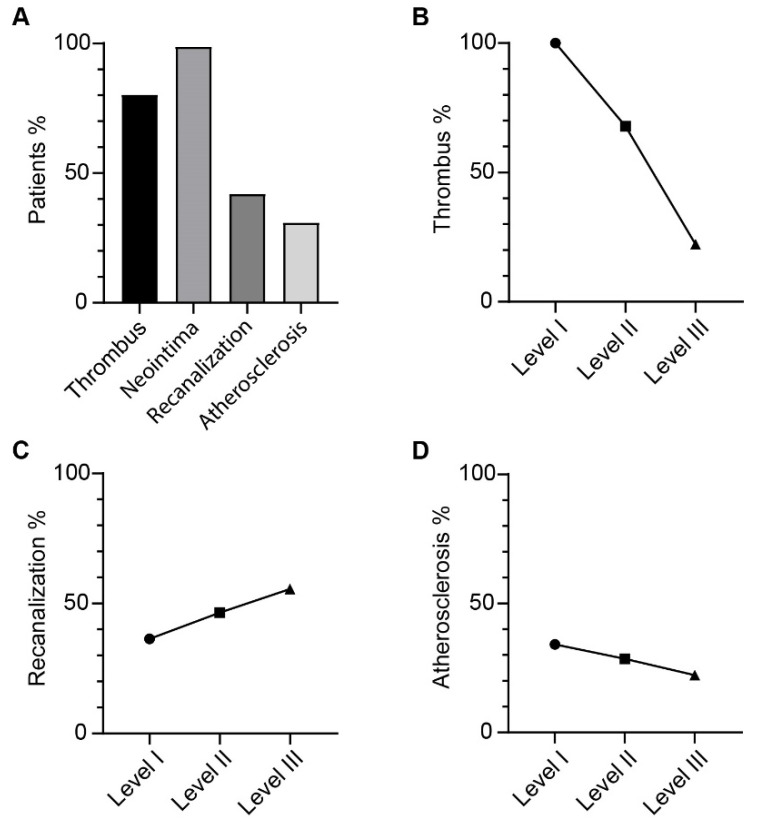
Distribution of different lesions in the CTEPH patient population. (**A**) Percentage of CTEPH patients with thrombotic, neointima, recanalized, and atherosclerotic lesions in PEA specimens. Repartition of patients with thrombus (**B**), recanalization (**C**), and atherosclerosis (**D**) formation based on UCSD surgical classification.

Four basic lesions on the major pulmonary vascular bed were carefully observed and classified as fresh/organized thrombi, neointima, atherosclerosis, and recanalized regions following the previous report [7]. Thrombotic lesions marked by CD61 were observed in 65 samples (80.24%), composed mainly of fibrin and platelet aggregates [12], with a small amount of fresh area rich in erythrocytes located in the center of the proximal lumen (Figure 1A). Neointima formation was noticed in almost all patients (*n* = 80, 98.77%), which was characterized by the accumulation of α-smooth muscle actin (α-SMA)-positive cells (Figure 1B), including myofibroblasts, dedifferentiated pulmonary artery smooth muscle cells (PASMC), or cells derived from endothelial-to-mesenchymal transition (EndoMT) [13,14]. Recanalization was observed in 34 cases (41.98%), presenting either as capillary-like neo-vessels in collagen-rich neointima or as irregular muscular-type small arteries in obstructed distal areas (Figure 1C). Atherosclerotic lesions characterized by macrophage-derived foam cells and the accumulation of cholesterol crystals were found in 30.86% (*n* = 25) of all patients (Figure 1D). It was interesting to find that, in some cases, thrombi were observed directly on top of atherosclerotic plaques (Figure S2 in the Supplementary Materials). Thrombi and atherosclerosis were dominant in UCSD level I PEA materials, and a decreasing trend was observed from proximal (UCSD level I or II) to distal (UCSD level III) types of diseases, whereas recanalized neo-vessels were more frequently observed in UCSD level III cases (Figure 2). 

**Figure 3:**
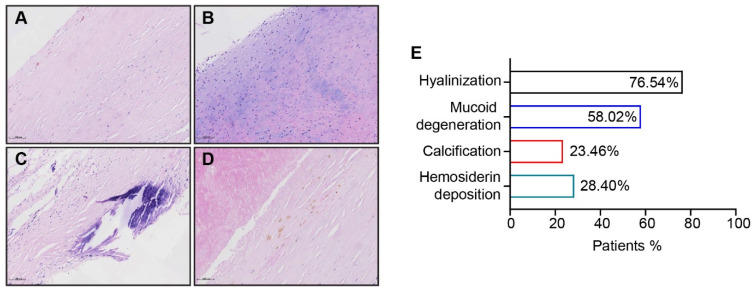
The remodeling of the vascular extracellular matrix in PEA-removed tissues from CTEPH patients. (**A**–**D**) Representative images of hyalinization (**A**), mucoid degeneration (**B**)**,** calcification (**C**), and hemosiderin deposition (**D**) in HE staining. (**E**) Distribution of ECM alteration in the CTEPH patient population. Scale bar = 100 µm.

In addition to the four basic lesions, we also noted the remodeling of the vascular extracellular matrix (ECM) in PEA-removed materials: hyalinization was observed in 62 patients’ specimens (76.54%), presenting as a homogeneous distribution of collagen fibers (Figure 3A); mucoid degeneration was seen in 47 cases (58.02%, Figure 3B), calcification was found in 19 cases (23.46%, Figure 3C), and hemosiderin deposition was identified in 23 cases (28.40%). All were degenerative changes of the pulmonary arterial wall following long-standing pulmonary hypertension.

### 3.3. Atherosclerotic Lesion Formation Corresponds with Main Pulmonary Artery Dilation in CTEPH Patients 

**Figure 4:**
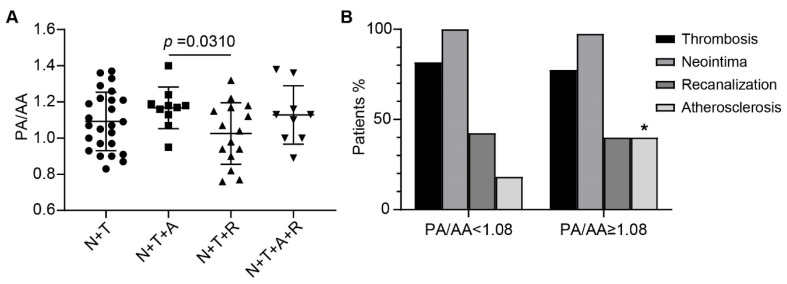
Association between atherosclerotic lesion formation and main pulmonary artery dilation in CTEPH. (**A**) In the presence of neointima and thrombi, the mean PA/AA is higher in cases with atherosclerotic lesions than those with recanalized neo-vessels. N, neointima, T, thrombus, A, atherosclerosis, R, recanalization. (**B**), Patients with PA/AA ≥ 1.08 were more frequently identified with atherosclerotic lesions. Data are presented as the mean ± SD. * *p* < 0.05. Student’s *t*-test for (**A**) and chi-square test for (**B**).

As a long-term consequence of elevated PAP, pulmonary artery dilation has been recognized as a common feature of PAH and CTEPH [15]. The ratio of main pulmonary artery diameter/ascending aorta diameter (PA/AA) measured by computed tomographic pulmonary angiogram has been reported to be utilized to evaluate PA dilation in CTEPH patients [16]. In our cases, with the presence of both neointima (N) and thrombi (T), higher PA/AA ratios were observed in cases with atherosclerotic lesions (A) compared to those without atherosclerosis and with recanalized neo-vessels (R) instead (N + T+A vs. N + T+R). A similar trend was also noticed in groups of N + T + A vs. N + T and N + T + A + R vs. N + T + R, although without statistical significance (Figure 4A). Moreover, atherosclerotic lesions were more frequently observed in CTEPH patients with higher PA/AA ratios (40% vs. 18.18%, cutoff value = 1.08, *p* = 0.0432; Figure 4B). However, there was no evident difference between the two groups regarding their sex, BMI, total cholesterol (TC) and triglyceride (TG) levels, and the prevalence of smoking history and atherosclerotic diseases (including coronary artery disease, carotid stenosis, and lower limb atherosclerosis) (Table S1 in the Supplementary Materials). These data indicate a potential association between atherosclerosis and dilated pulmonary arteries in CTEPH patients.

### 3.4. Patients with Complex Lesions have Greater Haemodynamic Improvement after PEA

**Figure 5:**
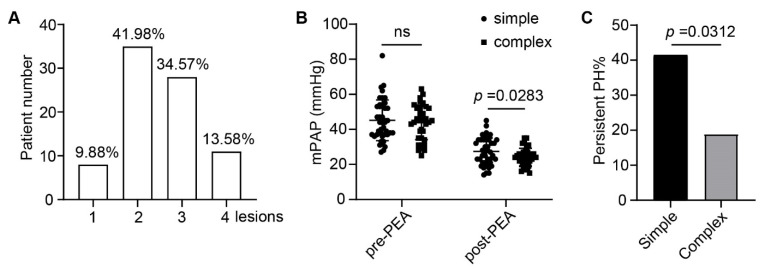
Correlation between lesion type and hemodynamic parameters of patients with CTEPH. (**A**) Number of CTEPH patients with one to four types of lesions. (**B**) mPAP before and after PEA procedure in patients with simple (one to two types) and complex (three to four types) lesions. (**C**) Percentage of persistent pulmonary hypertension (mPAP ≥ 30 mmHg) after PEA in patients with simple and complex lesions. Data are presented as the mean ± SD or percentage. Student’s *t*-test for (**B**) and chi-square test for (**C**).

Eight specimens (9.88%) in our study were identified with only neointima or thrombus formation, while most cases were observed with more than one type of the four basic lesions (Figure 5A). However, there was no difference in preoperative or postoperative mPAP among patients with only a single type or more than one type of lesions (Figure S4 in the Supplementary Materials). We then divided the combination of different types of lesions into simple (one to two types, 51.85%) and complex (three to four types, 48.15%) lesions. Interestingly, although the mean mPAP levels were similar between the two groups before surgery, patients with complex lesions had lower postoperative mPAP (Figure 5B). Persistent pulmonary hypertension (mPAP ≥ 30 mmHg after PEA) was observed in 30.77% of all patients by right heart catheterization. Similarly, after surgery, a lower percentage of persistent PH was noted in cases with complex lesions (18.92% vs. 41.46%, *p* = 0.0312, Figure 5C). The above data indicate that patients with complex lesions may benefit more from PEA.

## 4. Discussion

In the present study, we described the histopathological characteristics of the endarterectomized specimens from patients with CTEPH and analyzed the association between thromboembolic vasculopathy and pulmonary hypertension. The incidences of the four basic lesions were comparable with the report from Quark et al. [7]. Neointima formation was found in almost all cases; thrombus and atherosclerosis were predominantly present in proximal diseases (UCSD classification levels I and II), while recanalized neo-vessels were more frequently observed in distal pulmonary arteries, indicating an important role of the varying anatomical and hemodynamic conditions during the evolution of different kinds of lesions in CTEPH. Collagen deposition and calcification were identified in the obstructed areas of pulmonary arteries, both of which were degenerative changes of the extracellular matrix due to pulmonary hypertension and aging.

Atherosclerotic lesions containing foam cells and cholesterol crystals were first noted to present in CTEPH vascular beds by Bernard and Yi in 2007 [6]. Later in 2017, Kivrak et al. suggested an increased prevalence of atherosclerosis in CTEPH patients [17]. In concordance with this report, our data showed a 30.86% incidence rate of atherosclerosis in the study population. No difference was identified between patients with or without atherosclerotic lesions regarding the general cardiovascular disease risk factors. As the emergence of pulmonary arterial atherosclerotic lesions is not correlated with systematic symptomatic atherosclerosis, future studies are required to clarify the possible mechanisms. It was interesting to find higher PA/AA levels in CTEPH patients with atherosclerosis within pulmonary arteries in our study cohort. A further assessment revealed that most atherosclerotic lesions were located in the proximal portion of the pulmonary artery. Moreover, we noticed that 88% of the atherosclerotic plaques were accompanied with thrombi (vs. 12% without thrombi, data not shown), which is in agreement with Bernard and Yi’s data, where collections of foamy histocytes and cholesterol clefts were found to be associated with fibrinous materials [6]. Although several studies have reported associations between thromboembolism and atherosclerosis [18], the underlying mechanism remains largely unknown. It is reasonable to infer that the deposition of foam cells and cholesterol crystals might be a secondary alteration following thromboembolism. On the other hand, the exposure of the thrombogenic necrotic core in blood flow would directly lead to the propagation of in situ atherothrombosis through the coagulation cascade [19,20].

Our data showed that the removal of obstructed pulmonary materials with three or more types of lesions was associated with lower postoperative mPAP, although no difference was noticed at the baseline. We then noticed that distal disease (UCSD classification level III) was less frequently found in cases with complex lesions than simple lesions (5.13% vs. 16.67%, *p* = 0.0987 by the chi-square test, data not shown). Based on this fact, a potential explanation for the discrepancy in hemodynamic changes between patients with simple or complex lesions may be the inaccessibility of occlusions further than segmental arteries in cases with distal thromboembolic diseases. Further investigation is required to elucidate how specific types of lesions would contribute to the maintenance of pulmonary hypertension in CTEPH. 

Previous studies have demonstrated that the presence of histopathological recanalized lesions is a predictor of better hemodynamic improvement and lower mortality in short- and long-term follow-ups after PEA [7,9]. In our study population, a tendency of lower postoperative mPAP (24.73 ± 5.30 mmHg vs. 26.71 ± 7.69 mmHg, *p* = 0.2059 by the *t*-test, data not shown) was noticed in cases with recanalization, although without statistical significance. In addition, recanalized lesions were less frequently identified in patients with persistent pulmonary hypertension (29.63% vs. 48.15%, *p* = 0.1114 for the chi-square test, data not shown). These data are consistent with earlier reports, and we presume that the non-significance may be due to the wide variation in postoperative hemodynamic parameters.

In our study, the mean time from the onset of PH symptoms to PEA was much longer than what was reported based on European registries [21], and this may result from complicated technical and economic factors. Firstly, PEA was not performed in China until 2009, almost 20 years after its emergence. Secondly, the number of CTEPH centers qualified for PEA in China is still far less than that in Europe, considering the substantial patient population. In addition, the unbalanced development of the economy and medical system increased the difficulty for many Chinese CTEPH patients to seek further medical attention other than anticoagulant treatment [22]. These reasons may also partly explain the fact that only 81/272 (29.78%) of all consulted CTEPH patients chose PEA.

## 5. Limitations

The current study described the end-stage histopathological characteristics of major pulmonary arteries in CTEPH without following the sequence of events from early thrombus, as pulmonary endarterectomized tissues only provided a cross-sectional view of the late changes of CTEPH. Therefore, further studies are required to investigate the pathophysiology of this thromboembolic disease. 

Next, we are aware that, although we demonstrated the correlation between histological features and clinical manifestations, the evidence was not strong enough to establish casual relationships. In addition, histopathological examinations can only provide a descriptive analysis after PEA was accomplished. Based on these facts, the present results scarcely have an impact on the optimal therapeutic choices in the treatment of CTEPH and cannot help the multidisciplinary team (MDT) to decide which group of patients would benefit more from PEA. However, we believe that our findings may provide valuable bases for further studies, which may contribute to elucidating the underlying pathophysiology of CTEPH.

Furthermore, a long-term follow-up study after patients were discharged from the hospital was not provided. As Corsico et al. demonstrated that, after surviving the perioperative period, the early hemodynamic benefits of PEA remained largely unchanged over 5 years [23], and as the sample size of our study cohort was reliable for statistical analysis, it is reasonable to assume that the hemodynamic results and survival at discharge would, to a large extent, reflect patients’ pulmonary vascular conditions within 5 years. The median follow-up time of our study cohort was 2.05 years (range from 0.66 to 4.96 years), and additional efforts deserve to be made at our center to explore long-term survival and recurrence. 

## 6. Conclusions

In summary, we described the detailed histological characteristics of CTEPH and revealed innovative associations between pulmonary vasculopathy and clinical manifestations. Our data provided the first evidence suggesting that atherosclerotic lesion formation is correlated with pulmonary artery dilation in CTEPH. Furthermore, we showed that the removal of occluded tissues composed of complex lesions is related to lower postoperative mPAP. Further studies are needed to address the nature of this association and evaluate its implications for the clinical management of CTEPH.

## Figures and Tables

**Table 1:** Study population characteristics.

Parameters	Case Number
Subjects, *n*	81
age at PEA years	53 (41–61)
Sex male	54 (66.67%)
BMI (kg·m^2^)	24.38 ± 3.53
Disease duration (year)	2.17 (0.92–5)
History of acute PE/DVT	59 (72.84%)
Thrombophilia	8 (9.88%)
WHO functional class I~II WHO functional class III~IV	28 (34.57%) 51 (62.96%)
6MWD (m)	379 ± 100
SvO_2_%	68.38 ± 11.25
use of PH targeting drugs	44 (54.32%)
pre-/post-operative mPAP (mmHg)	45 ± 11/26 ± 7
pre-/post-operative PVR (dynes·sec^−1^·cm^−5^)	937 ± 414/470 ± 172

Values are expressed as median with first and third quartiles (Q1–Q3), *n* (%), or means ± standard deviations (SDs), unless otherwise indicated. BMI: body mass index; PE: pulmonary embolism; DVT: deep vein thrombosis; 6MWD: 6-min walk distance; PH: pulmonary hypertension; mPAP: mean pulmonary artery pressure; PVR: pulmonary vascular resistance.

## Data Availability

The datasets generated and/or analyzed in the current study are available from the corresponding author upon reasonable request.

## Supplementary Materials

The following supporting information can be downloaded at: https://www.mdpi.com/article/10.3390/jcm11226659/s1, Figure S1:Representative gross photographs of different types of PEA specimens by Jamieson surgical classification.; Figure S2:Representative images of HE staining and CD61 immunohistochemical staining of atherosclerotic lesion accompanied with thrombus.; Figure S3: Representative CTPA image of CTEPH.; Figure S4:mPAP levels were comparable among patients with one type and with several types of lesions before and after PEA.; Table S1: Clinical characteristics of patients with or without atherosclerosis.

## Nonstandard Abbreviations

CTEPH	chronic thromboembolic pulmonary hypertension
PEA	pulmonary endarterectomy
BPA	balloon pulmonary angioplasty
PH	pulmonary hypertension
PA/AA	ratio of pulmonary artery diameter to ascending aorta diameter
mPAP	mean pulmonary arterial pressure
PE	pulmonary embolism
DVT	deep vein thrombosis
PVR	pulmonary vascular resistance
mPCWP	mean pulmonary capillary wedge pressure
CO	cardiac output
ECM	extracellular matrix
