# Supplementary material for: Clinicopathological Correlation of Chronic Thromboembolic Pulmonary Hypertension: A Retrospective Study
Source: J Clin Med. 2022 Nov 10;11(22):6659. doi: 10.3390/jcm11226659 (PMC9694879; doi:10.3390/jcm11226659)
Supplement: Supplementary file 1 [file jcm-11-06659-s001.zip › jcm-1976754-supplementary.pdf]

**Supplementary Materials**

**For**

# Clinicopathological Correlation of Chronic Thromboembolic Pulmonary Hypertension: A retrospective study

**Manuscript ID: jcm-1976754**

**Contents:**

Figures S1 through S4 and Figure Legends

Table S1

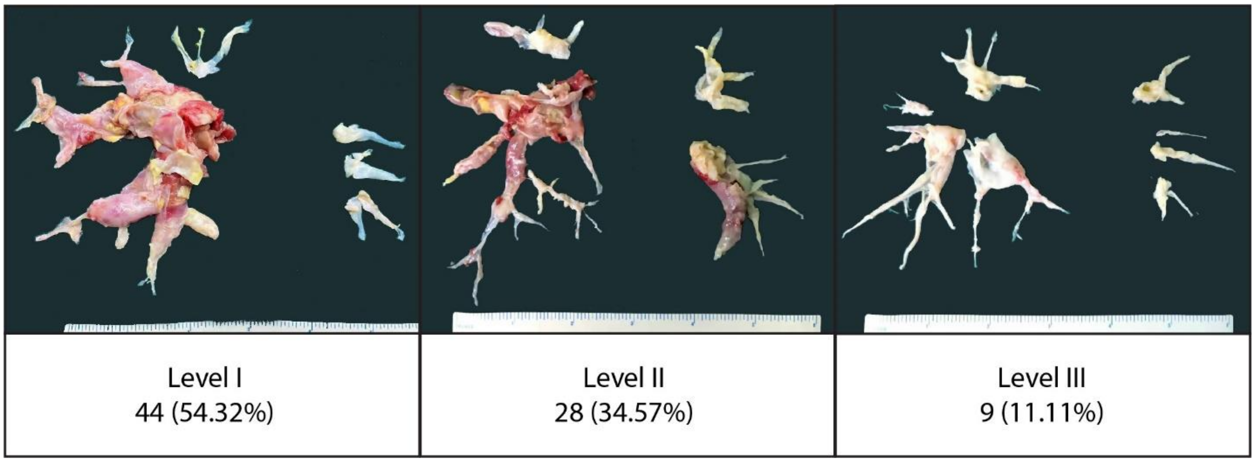


**Fig. S1.** Representative gross photographs of different types of PEA specimens by UCSD surgical classification.

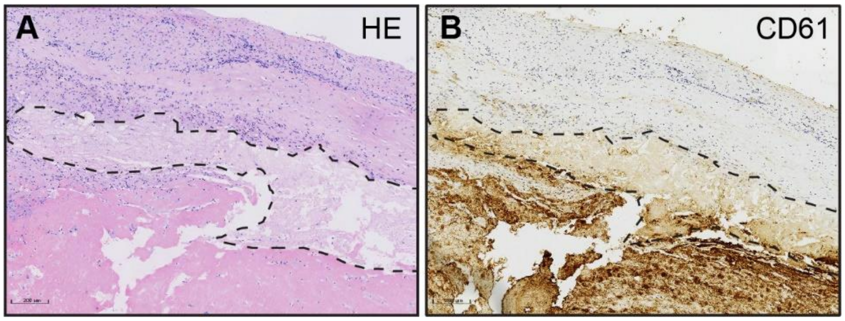


**Fig. S2.** Representative images of HE staining (A) and CD61 immunohistochemical staining (B) of atherosclerotic lesion accompanied with thrombus. Necrotic plaques were indicated by dashed lines. Scale bar =200 μm.

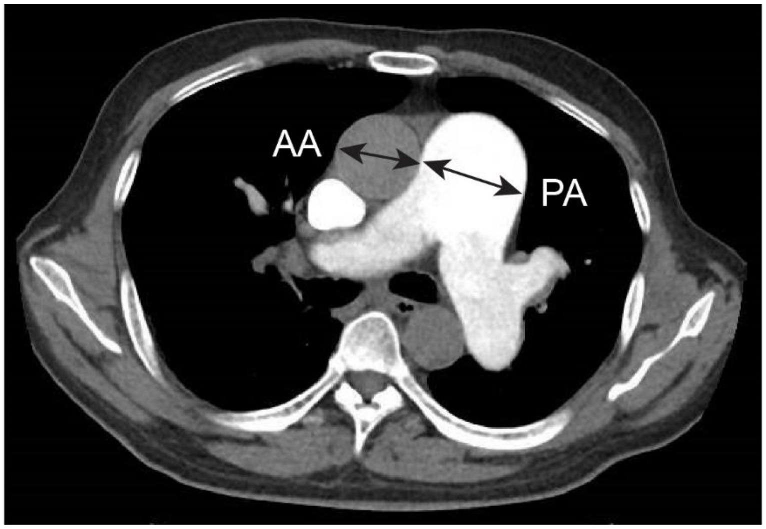


**Fig. S3.** Representative CTPA image of CTEPH. Pulmonary artery diameter (PA) and ascending aorta diameter (AA) were measured at the level of the bifurcation of the pulmonary trunk to calculate PA/AA.

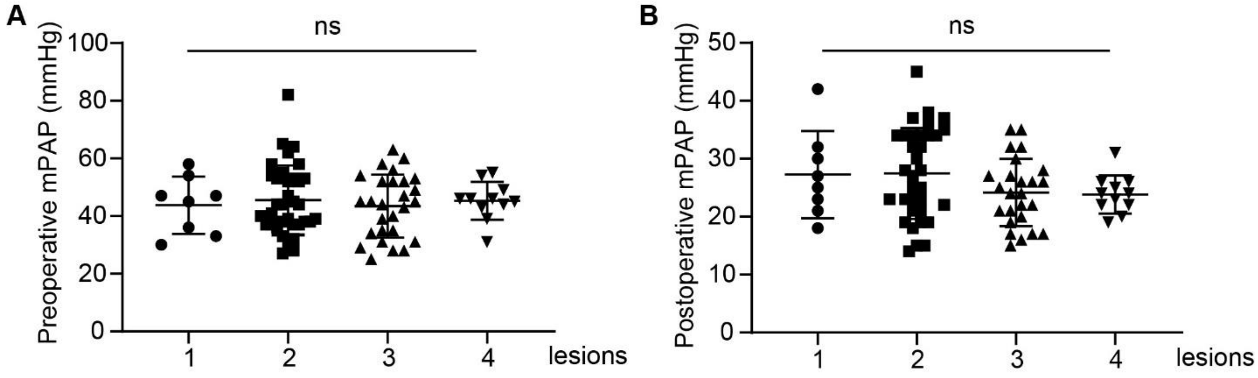


**Fig. S4.** mPAP levels were comparable among patients with one type and with several types of lesions before (A) and after PEA (B). Data are presented as mean ± SD and analyzed through one-way ANOVA.

**Table S1.** Clinical characteristics of patients with or without atherosclerosis.

| Parameters | Non-atherosclerosis | Atherosclerosis | *p*-value |
|---|---|---|---|
| Subjects n | 56 | 25 | |
| Age | 49.14±13.27 | 53.44±10.47 | 0.1564 |
| Sex male% | 69.64 | 60 | 0.3951 |
| BMI | 23.90±3.14 | 25.42±4.12 | 0.0743 |
| Ever smoking% | 33.93 | 36 | 0.8563 |
| Atherosclerotic diseases%* | 30.36 | 40 | 0.3951 |
| TC (mmol/L) | 4.04±1.12 | 3.86±1.40 | 0.5714 |
| TG (mmol/L) | 1.47±0.80 | 1.29±0.96 | 0.4153 |

Values are expressed as n (%) or means ± standard deviations (SDs) unless otherwise indicated. BMI: body mass index; TC: total cholesterol; TG: triglyceride. *Atherosclerotic diseases include coronary artery disease, carotid stenosis, and lower limb atherosclerosis.
